# How types of premises modulate the typicality effect in category-based induction: diverging evidence from the P2, P3, and LPC effects

**DOI:** 10.1038/srep37890

**Published:** 2016-12-16

**Authors:** Xiuling Liang, Qingfei Chen, Yi Lei, Hong Li

**Affiliations:** 1 Institute for Advanced Study, ChengDu University, ChengDu, 610106, China; 2 Research Centre for Brain Function and Psychological Science, Shenzhen University, Shenzhen, 518060, China; 3 China center for special economic zone research, Shenzhen University, Shenzhen, 518060, China

## Abstract

Behavioural studies have indicated that semantic typicality influences processing time and accuracy during the performance of inductive reasoning (*i.e*., the typicality effect). The present study examines this effect by manipulating the types of premises and conclusions (*i.e*., general, typical, or atypical) at an electrophysiological level using a semantic category-based induction task. With regard to behavioural results, higher inductive strength was found in typical conclusions in all premise conditions, whereas a longer response time for atypical conclusions was only found in general and typical premise conditions. The ERP results had different response patterns: in the general premise condition, a larger P2, as well as a smaller P3 and LPC (500–600 ms), were elicited by atypical conclusions relative to typical ones; in the typical premise condition, a larger P2 and LPC (600–700 ms) were found for atypical conclusions; in the atypical premise condition, however, only a larger P2 was found for atypical conclusions. The divergent evidence for the typicality effect indicated that the processing of the typicality effect in general, and specific premise conditions, might involve different cognitive processes, such as resource allocation and inference violation, which yielded new insights into the neural underpinnings of the typicality effect in a category-based induction.

The ability to use semantic concepts representing semantic categories to name, learn, classify, and reason is crucial in our daily lives, however, not all members of a category are of equal importance, and certain categories members (*e.g*., sparrow, crow) that are more typical may be more representative than others (*e.g*., penguin, ostrich). Furthermore, typicality has a significant effect on processing time and accuracy during object naming, learning, categorisation, and reasoning^1^,^2^,^3^. For example, the verification of a category member was faster if the member was a typical item than if it was an atypical item^4^. This is known as the typicality effect, which has been examined in many semantic tasks, such as category or picture naming^5^, category-based verification or reasoning^3^,^6^,^7^, and reading and sentence production^8^,^9^. Unfortunately, the neural underpinnings of the typicality effect remain poorly understood. To address this issue, the present study gathers evidence from event related potentials (ERPs) using a category-based induction task.

The evidence of the typicality effect in cognitive neuroscience was mainly based on several ERP studies, such as that seen during category-based verification, in which participants were required to judge whether, or not, the stimulus belonged to a given category^3^,^10^,^11^. For example, the ERP amplitude (300–450 ms) elicited by atypical words was more negative than typical words during a category verification task^10^. Furthermore, when performing a category verification task via pictorial stimuli, a shorter P3 latency-to-peak was elicited by typical items relative to atypical ones^11^. Recently, Räling and colleagues^3^ found that N400 amplitude elicited by atypical congruent targets was larger than typical congruent targets when examining the influence of typicality on semantic processing during an auditory category member verification task. These results suggested that the P3 or N400 component was modulated by typicality in category verification paradigms.

A similar typicality effect was found in other tasks, such as category-based deduction, semantic categorisation, and lexical decision^6^,^12^,^13^,^14^. For example, when participants were required to decide whether, or not, an incoming stimulus (conclusion) had the property described in the category presented before (premise), reaction time data revealed a significant typicality effect, and ERP data indicated that larger N1, P2, and N400 amplitudes were elicited by atypical words relative to typical words^6^. Recently, Wang and colleagues^15^ explored the typicality effect via different a categorisation method: that of either inclusion or exclusion. Specifically, after being presented with six consecutive words that shared one feature, participants were required to judge whether the seventh word also possessed that feature (*i.e*., inclusion), or whether it did not. They found that a larger P2 and smaller N400 were elicited by typical members relative to atypical members in inclusion, whereas typical members elicited a larger N2 than atypical members in the exclusion^15^. These results further indicated that the typicality effect has a pervasive influence on the early perceptual and attention processes, as well as later semantic processes and categorisation.

In fact, Lei *et al*.^6^ mainly explored the typicality effect during category-based deduction, in which the category in the premise included the category in the conclusion (e.g., Birds have property X; → Sparrows/penguins have property X; The bird includes the sparrow and penguin): as a result, the argument is logically valid^16^,^17^,^18^, yet, little is known about the neural basis of the typicality effect during category-based induction, in which the category in the premise belongs to the category in the conclusion (e.g., Sparrows/penguins have property X; → Birds have property X), or both premise and conclusion belong to same basic level category (e.g., Sparrows/penguins have property X; → Chicken/crows have property X; Both of them are birds). Furthermore, the method in Wang *et al*.^15^ was also different from category-based induction in essence. Before testing, specifically, participants were required to repeat category learning and classification until the accuracy reached 90%. In the test, participants were explicitly told that: “All members of this category possess a feature X, whereas non-members of this category do not possess this feature.” As result, this task is more likely to be a category-based verification, rather than category-based induction. Accordingly, further studies were needed to explore the effects of typicality on a category-based induction task via ERPs.

Based on Lei *et al*.^6^, and using a simplified category-based deduction task (e.g., Bird has property X; → Sparrow), Long *et al*.^19^ used a similar method to explore semantic category-based induction, in which the premise was represented by a category member and a blank property linked by a blank space (e.g., Apple X1, which means that the apple has property X1), while an additional “?” was shown at the end of the conclusion (e.g., Banana X1?, which means that participants were required to determine whether or not the conclusion was acceptable). As the authors said, this way of presenting reasoning tasks had certain advantages (e.g., reducing the effect of prior knowledge), and the present authors think that it is more appropriate to investigate the neural basis of the typicality effect. Although several ERP studies explored the time course of category-based induction^19^,^20^, little is known about the neural basis of typicality effect when performing category-based induction. Accordingly, we set out to explore this issue using ERPs.

As mentioned above, previous studies revealed that N1, P2, N2, P3, and N400 mean amplitudes or peak-latencies were sensitive to the typicality effect. Indeed, N1 was sensitive to selective attention, which influenced the further processing of perceptual features^21^,^22^. Following N1, the P2 effect reflected the higher-order perceptual processing, which was modulated by attention^23^,^24^,^25^. For example, when participants were required to make a “high” or “low” similarity judgment for the subsequently presented geometric shapes, a larger P2 was elicited by stimuli with a feature difference^23^. Recent studies found that the P2 was also sensitive to semantic processing and language^26^,^27^. For example, a larger P2 was elicited by Chinese characters with low combinability and consistency relative to characters with high combinability and consistency^26^.

Furthermore, previous studies have shown that the N2 effect was modulated by perceptual mismatching, which has been extended into a reasoning task^28^,^29^,^30^. For example, the minor premise in conditional arguments (affirmation of the consequent: If P then Q; Q//therefore P) prompted participants to produce an N2 wave related to the violation of expectations^29^. After N2, the parietal P3 was typically believed to reflect an information-processing cascade related to attentional and memory mechanisms^23^,^31^,^32^. That is, the P3 amplitude was relatively large when tasks were undemanding, whereas more difficult demands usually reduced P3 amplitude^32^,^33^. Recent studies about reasoning indicated that P3 amplitudes reflected the satisfaction of expectations^19^,^34^,^35^,^36^,^37^. That is, the P3 effect elicited by matched arguments was larger than that for mismatched ones. For example, Long *et al*.^19^ found a larger P3 amplitude for logically related arguments (Apple X1 → Banana X1?) relative to arguments that the premises were unrelated to the conclusions (Apple X1 → Telephone X1?).

Moreover, late ERP components, such as N400 and LPC, were also found for the processing of typical effect or category based-induction. Previous studies have found that the N400 effect was sensitive to the strength of semantic relations^38^,^39^,^40^. In priming studies, for example, a larger N400 effect was elicited by unrelated or less-related words relative to related words^41^. During reasoning, the LPC (*c*. 600 ms) was modulated by different factors in both EEG and MEG studies, such as mismatched conclusion or property violations^19^,^35^,^37^. For example, a larger LPC at 700–800 ms was elicited by conclusions with unrelated properties relative to conclusions with related properties^19^. In fact, previous studies have found that the LPC amplitude was sensitive to old/new effect^42^, contextual effect^43^, processing manipulation^44^,^45^, as well as decision accuracy and confidence^46^. For example, a larger LPC was elicited by more deeply encoded items, or words attracting correct recognition decisions.

Based on the aforementioned considerations, the main purpose of this study was to explore how types of premises modulated the typicality effect in category-based induction at behavioural and electrophysiological levels. To achieve this, three types of premises and conclusions (general, typical, and atypical) are manipulated. In the general premise condition, participants reason from basic level category to general, typical or atypical members of the category (e.g., Bird X → Bird/sparrow/penguin X?). In the typical premise condition, they reason from typical premises to general, typical or atypical members (e.g., Crow X → Bird/sparrow/penguin X?). In the atypical premise condition, participants reason from atypical premises to general, typical or atypical conclusions (e.g., Ostrich X → Bird/sparrow/penguin?). Participants are required to assume the that information about the premise is true (e.g., Crow has property X), and to assess whether, or not, the information about the conclusion (e.g., Penguin has property X) is plausible, and make a “strong” or “not strong” response, which has been used extensively^17^,^18^.

The key focus here was on the electrophysiological data elicited by the conclusion (s), upon which certain judgments could be made. Based on previous studies, short RTs and higher “strong” responses should be found for typical conclusions relative to atypical conclusions^1^,^9^. In the field of ERP study, typicality has a pervasive influence on category-based verification and reasoning, which reflects on early N1 and P2 effects related to perceptual and attention processes, and later P3 or N400 effects related to semantic processes and categorisation^6^,^10^,^11^,^15^. Accordingly, significant differences in the ERP indices should be found between typical and atypical members. For example, the P2 effect should be sensitive to the typicality effect in category-based induction, because the representativeness of typical and atypical conclusions was different. Furthermore, research on reasoning found a larger P3, or LPC, effect for matched items when performing conditional reasoning^35^,^36^, category-based induction^19^, or transitive inference^37^. In the present study, a larger P3 or LPC amplitude should be elicited by typical conclusions, because they tend to match the premises to a greater extent^47^,^48^.

More importantly, the types of premises should have a critical regulatory role in the typicality effect when exploring the ERP effects elicited by the conclusions, especially for general premises and specific (typical and atypical) premises. In the general premise condition, specifically, the category in the premise includes, or is identical to, the category in the conclusion (*e.g*., Bird X; → Bird/sparrow/penguin X?), so if the information about the premises is true, the information about the conclusions is also true. For example, if all birds have property X is true, sparrows must have property X, because a sparrow is a type of bird; however, in the typical/atypical premise condition, the information about the conclusions is not necessarily true, but only has a “strong” or “not strong” plausibility. For example, if all sparrows have property X is true, all birds/penguins do not necessarily have property X. Furthermore, different response patterns should also be found in typical and atypical premises, because typical premises are more representative than atypical members, especially when the conclusion is a general category. As results, although early ERP effects related to the perceptual and attention processes for typical and atypical conclusions should be found in different premise conditions (*e.g*., P2), the late ERP effects related to premise-conclusion matching or categorisation violations should have different response patterns (*e.g*., P3 or LPC).

## Methods

### Participants

15 healthy undergraduate students (nine female) between the ages of 19 and 24 were paid for participating in the main experiment. Participants that initially rated the stimuli did not participate in the main procedure. All participants were healthy and right-handed with normal or corrected-to-normal vision, and gave their informed written consent before the experiment. All experimental protocols were approved by the University’s ethics committee (The Medicine Medical Ethics Committee of Shenzhen University), and the methods were carried out in accordance with the relevant guidelines and regulations.

### Stimuli

Before the main experiment, six natural categories (*i.e*., birds, fishes, insects, vegetables, flowers, and fruits) and four artificial categories (*i.e*., clothing, musical instruments, furniture, and tools) were selected as stimuli. In a manner similar to previous studies^6^,^14^, forty undergraduates were required to write members of the above categories as much as possible.

After this, another 40 undergraduates participated in a typicality test, in which participants were required to estimate the extent to which these members epitomised the category on a seven-point scale, where 7 indicated the highest typicality. For example, category members such as “sparrow” and “crow” received a typical rating of “6” or “7” as birds. As a bird, however, the degree of typicality for category members such as “penguin” or “duck” was lower than that for “sparrow” and “crow”, and they might receive a rating of “3” or “4” as birds. After these studies, five typical members and five atypical members were selected from each category, plus these categories themselves, composed a set of 110 categories. An independent *t*-test suggested that there was a significant difference between typical members (*M* = 6.79, *SD* = 0.15) and atypical members (*M* = 4.65, *SD* = 0.52), *t* (58) = 21.66, *p* < 0.001.

The premise and conclusion of the arguments consisted of one of the above selected 110 categories or members (See Supplementary Information for S1 Table, S2 Table and S3 Table), as well as the blank property represented by capital letters ranging from A-Z (*e.g*., Sparrow A, which meant that sparrows have property A; see Table 1). The single premise category-based induction (*e.g*., Premise: Crows have property A; Conclusion: Sparrows have property A) was used to induce a memory load^1^,^19^. To reduce the effect of prior knowledge, a blank property (*e.g*., A) was used and never any actual properties as clarified further elsewhere^19^,^49^. As mentioned earlier, the types of premises (general, typical, or atypical) and conclusions (general, typical, or atypical) were manipulated, and thus included nine main experimental conditions. Moreover, the identical conditions for typical and atypical members, as well as control condition, were regarded as baseline tasks. Specifically, the premise and conclusion for main experimental and identical conditions consisted of natural categories with the same blank property. For the control condition, however, the premise and conclusion consisted of natural and artificial categories respectively, and their properties were different.

As shown in Table 1, the general premise condition included three types of arguments: (1) General = General (G = G), the premise and conclusion consisted of the same category itself. (2) General-Typical (G-T), in which the premise consisted of a category and the conclusion consisted of a typical member; (3) General-Atypical (G-A), in which the premise consisted of a category but the conclusion consisted of an atypical member. Furthermore, the typical premise condition also included three types of argument: (1) Typical-General (T-G), in which the premise consisted of a typical member and the conclusion consisted of a category. (2) Typical-Typical (T-T), both the premise and conclusion consisted of typical members of the same category. (3) Typical-Atypical (T-A), the premise consisted of a typical member, while the conclusion consisted of an atypical member of the same category. Similarly, the atypical premise condition included three types of argument: (1) Atypical-General (A-G), in which the premise consisted of an atypical member and the conclusion consisted of a category. (2) Atypical-Typical (A-T), the premise consisted of an atypical member, while the conclusion consisted of a typical member of the same category. (3) Atypical-Atypical (A-A), both premise and conclusion consisted of atypical members of the same category. However, the baseline tasks were as follows: (1) Typical = Typical (T = T), the premise and conclusion consisted of the same typical members. (2) Atypical = Atypical (A = A), the premise and conclusion consisted of the same atypical members. (3) Control conditions, the premises were members of the natural category, while the conclusions were members of artificial categories.

### Procedure

In the main procedure, participants judged whether the argument was “strong” or “not strong” by pressing one of two keys. The order of the stimuli within the task was randomised and counterbalanced. Following previous studies^17^,^18^,^50^, the “strong” argument was defined as: Assuming that the information represented by the premises is true, this makes the information represented by the conclusions *plausible*.

**Figure 1:**
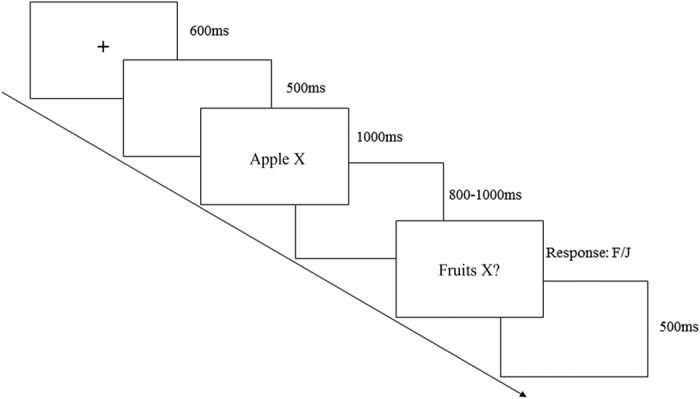
Illustration of the experimental procedure (general-typical condition).

The categories and category members were presented through one to three black Chinese characters, which appeared on a grey background as generated by the E-prime software. As shown in Fig. 1, a black fixation (“+”) was presented in the centre of the screen for 600 ms at the beginning of each trial, followed by a blank screen for 500 ms. Subsequently, the premise and a novel property linked by a blank space (*e.g*., Apple X) indicating that the category member had that property, was presented for 1000 ms, followed by a blank screen shown for a random duration (800–1000 ms). Next, the conclusion and a novel property ended in “?” (*e.g*., Fruits X?) linked by a blank space appeared and remained until participants made a response. Participants were instructed to respond as rapidly and accurately as possible to the conclusion, and make a “strong” or “not strong” response by pressing “F” or “J” with the left or right index finger, which was counterbalanced across subjects. The next trial began after the presentation of a blank screen for 500 ms.

To ensure that each participant understood the instructions, they were asked to repeat the instructions in their own words. Furthermore, to reduce the effect of familiarity, all categories and their members were presented by category at the beginning of experiment. The procedure was divided into practice and test phases. The tests phases consisted of 510 trials under the main experimental conditions (30 trials for G = G, 60 trials for each of the other conditions) and 240 trails for baseline conditions (60 trials for A = A, 60 trials for T = T, and 120 trials for the control condition). The identical condition (*i.e*., A = A, T = T) served as filler trials in which participants had to make “strong” responses, which was compared with the responses to control condition (“not strong”), to improve the efficacy of the design^51^. Furthermore, thirty practice trails were performed to familiarise participants with the procedure: these were selected from unused category members that were not included in the main experiment.

### ERP recordings and data analysis

Brain electrical activities were recorded from 64 tin electrodes mounted on an elastic cap based on the extended 10/20 system (Brain Products, GmbH, Germany; pass band: 0.05–100 Hz, sampling rate: 500 Hz). The ground electrode was on the medial frontal line and references were on the left and right mastoid^52^,^53^. The vertical and horizontal electro-oculograms (EOGs) were recorded from the left eye infra-orbitally and supra-orbitally, and the orbital rim of both eyes, respectively. All impedances were controlled to below 10 kΩ. All the bioelectric signals were analysed off-line using Brain Vision Analyzer 2.0. The signal was passed through a 0.1 to 35 Hz digital band-pass filter for off-line analysis. Ocular correction ICA was used to eliminate the artifacts such as blinks and eye movements. Off-line computerised artifact rejection was also used to eliminate trials with mean EOG (eye blinks and ocular movements), artifacts due to bursts of electromyography activity, amplifier clippings, or peak-to-peak deflections exceeding ± 80 μV.

**Figure 2:**
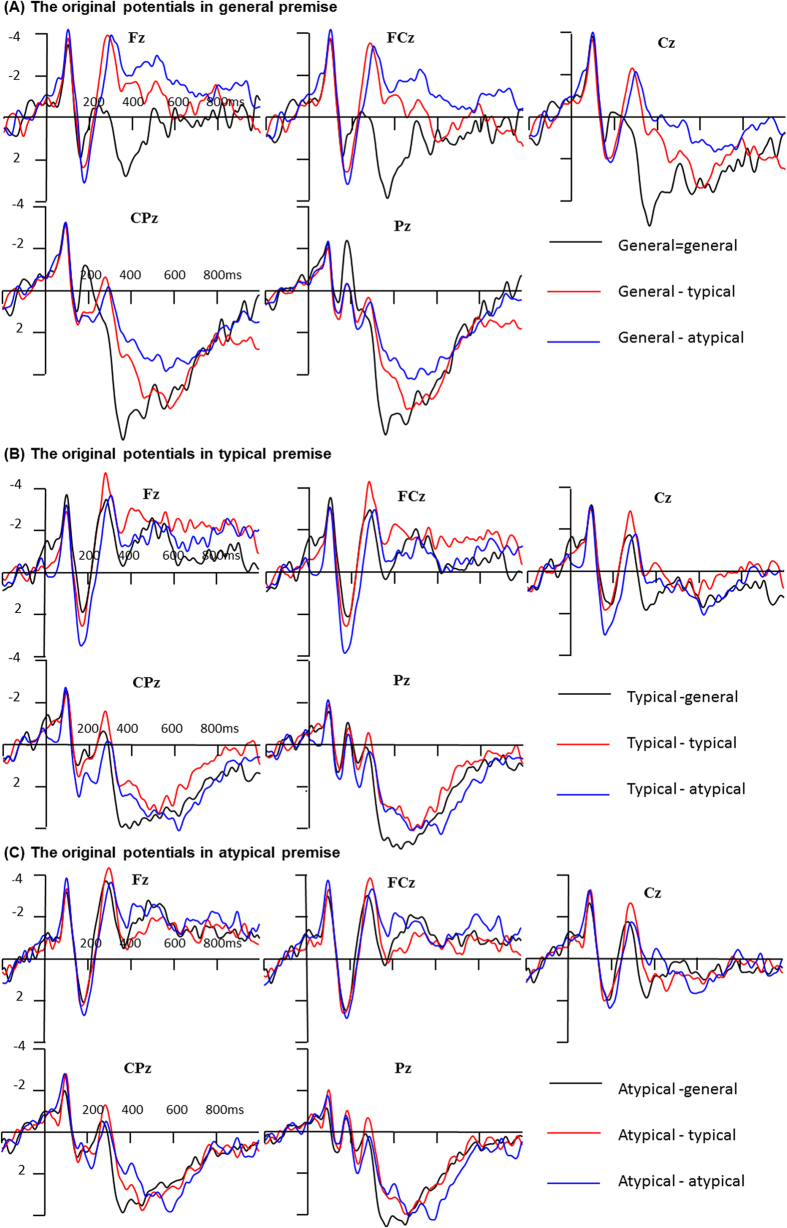
The ERPs elicited by different conclusions in general (**A**), typical (**B**), and specific premise condition (**C**).

In a manner similar to previous studies^6^,^19^, we focused on data elicited by the conclusion. As a result, epochs of 1000 ms, time-locked to the conclusion, including a 200 ms pre-stimulus baseline were extracted from the ongoing EEG, segmented and averaged (Fig. 2). In the present study, the identical condition (except for G = G), as well as the control condition, were regarded as baseline tasks to ensure an effective “strong/not strong” judgment^17^,^51^. In fact, participants made a very high “strong” response for the identical condition (*M* = 0.99, *SD* = 0.02), but made a very low response for the control condition (*M* = 0.08, *SD* = 0.25). However, including these conditions in the same analysis diluted any possible effect of typicality on induction. Indeed, we compared these identical conditions, and no significant ACC and RTs, as well as any ERP effects, were found among them.

Based on previous studies and the topographies, we focused our analysis on the mean peak latency, peak amplitude, and the mean amplitude between 50 ms and 150 ms (N1), 150 ms and 250 ms (P2), 250 ms and 350 ms (N2), 350 ms and 450 ms (P3), as well as the mean amplitude of LPC (500–600 ms, 600–700 ms, and 700–800 ms). Four-way repeated measures ANOVAs were performed for each time window, with premise (three types: general, typical, or atypical), conclusion (three types: general, typical, or atypical), laterality (three levels, left, middle, and right sites) and frontality (five levels, frontal: left—F3, middle—Fz, right—F4; frontal central: left—FC3, middle—FCz, right—FC4; central: left—C3, middle—Cz, right—C4; central parietal: left—CP3, middle—CPz, right—CP4; parietal: left—P3, middle—Pz, right—P4) as repeated factors. For all analyses, the degrees of freedom of the *F* ratio were corrected for violations of the sphericity assumption based on Greenhouse Geisser, and Bonferroni corrections were used for each comparison.

## Results

### Behavioural responses

Two-way repeated measures ANOVAs were performed for the proportion of “strong” responses and reaction times (RTs), with premise (three types: general, typical, or atypical) and conclusion (three types: general, typical, or atypical) as repeated factors. The main effect of premise on “strong” responses was significant, *F* (2, 28) = 7.12, *p* = 0.014, *η*^2^ = 0.34. Similarly, there was significant main effect on the conclusion, *F* (2, 28) = 8.82, *p* = 0.002, *η*^2^ = 0.39. In both situations, the proportion for atypical conditions (Premise: 0.68; Conclusion: 0.71) was lower than general (Premise: 0.94; Conclusion: 0.85) and typical conditions (Premise: 0.78; Conclusion: 0.82, *ps* < 0.02), whereas no significant difference was found between general and typical premises (*p* > 0.26). However, the interaction between premise and conclusion was not significant, *F* (4, 56) = 1.45, *p* = 0.25, *η*^2^ = 0.09.

The main effect of premise on RT was significant, *F* (2, 28) = 22.60, *p* < 0.001, *η*^2^ = 0.62. Similarly, there was significant main effect on the conclusion, *F* (2, 28) = 56.98, *p* < 0.001, *η*^2^ = 0.80. In both situations, RTs in the atypical conditions were longest (Premise: 945 ms; Conclusion: 806 ms), smallest in the general conditions (Premise: 818 ms; Conclusion: 992 ms), and intermediate in the typical conditions (Premise: 892 ms; Conclusion: 862 ms), which differed from each other for the three conditions (*ps* < 0.006). Furthermore, the interaction between premise and conclusion was significant, *F* (4, 56) = 3.33, *p* = 0.025, *η*^2^ = 0.19. Pair-wise comparison indicated that the RT for a general conclusion was shorter than typical and atypical conclusions in general, typical and atypical conditions (*ps* < 0.006). However, a shorter RT was found for typical conclusions relative to atypical conclusions only when the premise was of the general category (*p* < 0.001) and typical members (*p* < 0.05), but not for atypical premise members (*p* > 0.90).

### ERP results

**Figure 3:**
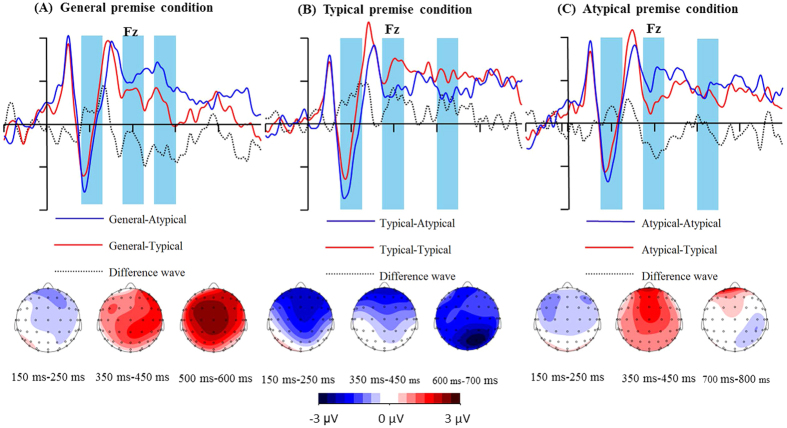
Difference waves and topographical maps for typical and atypical conclusions in general (**A**), typical (**B**), and atypical premise conditions (**C**).

As mentioned above, four-way repeated measures ANOVAs were performed for the mean peak amplitude, mean amplitude, and peak latency of N1, P2, N2, and P3, as well as the mean amplitude of LPC (see Tables 2 and 3, Figs 2 and 3).

### N1 (50–150 ms)

As shown in Tables 2 and 3, the main effect of the premise on the N1 peak was significant, the N1 elicited by a general premise was larger than that arising from an atypical premise (*p* = 0.016). The main effects of frontality and laterality on N1 mean amplitude were significant. Pair-wise comparison indicated the N1 component in frontal, frontal central and central sites was larger than central-parietal and parietal sites (*ps* < 0.05). The N1 at left and middle sites was larger than that at right sites (*ps* < 0.01). The interactions between laterality and premise on N1 mean amplitude and peak were significant. Pair-wise comparison indicated that the difference between a general premise and an atypical premise was found at middle and right sites (*ps* < 0.04), but not at left sites (*p* > 0.10). Although three way interaction among frontality, premise, and conclusion on the N1 amplitude was found, no significant interaction between premise and conclusion was found at sites with different frontality (*p*s > 0.10). No other significant difference was found.

### P2 (150–250 ms)

The main effects of conclusion on P2 mean amplitude and peak were significant. Pair-wise comparison found that the P2 peak elicited by general conclusion was smaller than typical and atypical conclusions (*ps* < 0.01), and the mean amplitude for general conclusions was smaller than that for atypical conclusions (*p* < 0.001). The interaction between premise and conclusion on P2 latency was significant, the P2 latency for general conclusions was shorter than that for atypical conclusion in the atypical premise condition (*p* = 0.03), but not in general and typical premise conditions (*ps* > 0.10).

The main effects of laterality on P2 mean amplitude and peak were significant. The P2 at right sites was larger than that at left sites (*p* = 0.03). Furthermore, the interaction between frontality and conclusion was significant. In addition to the larger P2 amplitude for atypical members relative to the general category found at all sites (*p*s < 0.04), the P2 elicited by atypical members was larger than that arising from typical members at frontal, frontal central, and central sites (*ps* < 0.05), but not at central parietal and parietal sites (*p*s > 0.33). Similarly, the interaction among frontality, laterality, and conclusion on P2 amplitude was significant. Further analysis indicated that a smaller P2 was elicited by general relative to atypical conclusions at Fz, F3, F4, FCz, FC3, FC4, and Pz (*p*s < 0.007), whereas such a difference between general and typical conclusions was only found at F3 sites (*p* = 0.013). Furthermore, the larger P2 elicited by atypical conclusions relative to typical conclusions was only found at F4 and FC4 (*p*s < 0.05). No other significant difference was found (*p*s > 0.05).

### N2 (250–350 ms)

The main effects of premise, as well as conclusion, on N2 peak and mean amplitude were significant. Pair-wise comparison indicted the N2 elicited by typical premise was larger than general premise (*ps* < 0.05). Similarly, the N2 elicited by typical and atypical conclusions were larger than general conclusion (*p*s < 0.02). The interaction between premise and conclusion on N2 mean amplitude was significant. The N2 elicited by a general conclusion was smaller than typical and atypical conclusions only in general premise conditions (*ps* < 0.01), but not in typical and atypical conditions (*ps* > 0.20). The interaction among frontality, premise, and conclusion on N2 mean amplitude was significant. Further analysis indicated that the smaller N2 elicited by a general conclusion relative to typical and atypical conclusions was only found at frontal, frontal central, and central sites (*p*s < 0.01). The three way interaction among laterality, premise, and conclusion on N2 mean amplitude and peak was significant. Further analysis found that the interaction between premise and conclusion was only found at middle sites (*p*s < 0.01), but not at left and right sites (*p*s > 0.10).

The main effects of frontality and laterality on N2 mean amplitude and peak were significant. The N2 component in frontal, frontal central sites was larger than central-parietal and parietal sites (*ps* < 0.05), and the N2 at left and middle sites were larger than that at right sites (*ps* < 0.001). The interaction between laterality and premise on N2 mean amplitude was significant. Pair-wise comparison indicated that the N2 elicited by a typical premise was larger than that of a general premise at middle (*p* = 0.03) and right sites (*p* = 0.01), but not at left sites (*p* = 0.15). Furthermore, the interactions between laterality and conclusion on N2 mean amplitude and peak were significant. That is, the N2 elicited by general conclusions was smaller than that from typical and atypical conclusions at middle and right sites (*ps* < 0.03), but not at left sites (*ps* > 0.24). The interaction among frontality, laterality, and conclusion was significant. Further analysis indicated that the difference between general and typical conclusions was found at Fz, F4, FCz, FC4, Cz, C4, CPz, CP4, Pz, and P4, whereas the difference between general and atypical conclusions was found at Fz, FCz, FC4, Cz, C4, CPz, CP4, Pz, P4, and P3 (*ps* < 0.05).

### P3 (350–450 ms)

The main effects of premise, as well as conclusion, on P3 peak and mean amplitude were significant. Pair-wise comparison indicted the P3 elicited by a general premise was larger than that from a typical premise (*p* < 0.05). Similarly, the P3 elicited by general conclusions was larger than typical and atypical conclusions (*p*s < 0.03). The interactions between premise and conclusion on P3 mean amplitude and peak were significant. Although the P3 elicited by a general conclusion was larger than that from typical conclusion in general and typical premise conditions (*ps* < 0.05), a larger P3 elicited by general conclusion relative to atypical conclusions was found in general and atypical premise conditions (*ps* < 0.05). Furthermore, the smaller P3 amplitude elicited by atypical conclusions relative to general and typical conclusions was only found in general premise conditions (*ps* < 0.02). The three interactions among frontality, premise, and conclusion on P3 amplitude and peak were significant. Further analysis indicated that the smaller P3 elicited by atypical conclusions relative to general (*p*s < 0.01) and typical conclusions (*p*s < 0.05) was only found in general premise conditions at frontal, frontal central, central, and central parietal sites. The three way interaction among laterality, premise, and conclusion on P3 amplitude was significant. Further analysis found that the interaction between premise and conclusion was more significant at middle (*p* = 0.001) and right sites (*p* = 0.003) relative to left sites (*p* = 0.03). No other significant difference was found.

The main effects of frontality and laterality on P3 amplitude and peak were significant. The P3 in central, central-parietal, and parietal sites was larger than that at frontal, frontal central sites (*ps* < 0.01), and the P3 at right sites was larger than left and middle sites (*ps* < 0.01). The interaction between laterality and premise on P3 amplitude was significant. Although the P3 elicited by a general premise was larger than that arising from a typical premise at all sites (*p* < 0.05), it was only larger than that arising from an atypical premise at middle sites (*p* = 0.025). Furthermore, the interactions between laterality and conclusion on P3 amplitude and peak were significant. That is, the P3 elicited by a general conclusion was larger than that from an atypical conclusions at all sites (*ps* < 0.03), whereas it was larger than that arising from typical conclusions only at middle and right sites (*ps* < 0.05). The three way interaction among frontality, laterality, and conclusion on P3 amplitude was significant. Further analysis indicated that the difference between general and typical conclusions was found at F4, FC4, C4, CPz, CP4, and P3, whereas the difference between general and atypical conclusions was found at Fz, F4, FCz, FC4, Cz, C4, CPz, CP4, and Pz (*ps* < 0.05).

### LPC (500–800 ms)

The main effect of premise on LPC was significant, pair-wise comparison found that the LPC elicited by a general premise was larger than that arising from typical and atypical premises at both 500–600 ms and 600–700 ms (*p*s < 0.03), whereas it was just larger than that from an atypical premise at 700–800 ms (*p* = 0.042). Furthermore, the interaction between premise and conclusion was significant at 500–700 ms, but not at 700–800 ms. At 500–600 ms, the mean amplitude of LPC elicited by atypical conclusion was smaller than that from a typical conclusion only in general premise condition (*p* < 0.03), but not in typical and atypical premise conditions (*p* > 0.50). At 600–700 ms, however, only larger LPC for atypical conclusion relative to typical conclusion was found in typical premise conditions.

The main effect of frontality, as well as laterality, was significant at 500–800 ms. The LPC component in central, central-parietal, and parietal sites was larger than frontal, and frontal central, sites (*p*s < 0.05). In addition, the LPC at right sites was larger than that at left and middle sites (*p*s < 0.01). Furthermore, the interaction between laterality and premise, as well as laterality and conclusion, was significant at 500–600 ms and 600–700 ms. The larger LPC elicited by general premise relative to typical and atypical premises was found in middle and right sites (*p* < 0.05), but not in left sites. Although the absolute difference among conclusions was larger at right sites relative to middle and left sites, they did not reach a significant level. The interaction between frontality and conclusion was significant at 600–700 ms and 700–800 ms. Further analysis indicated that the LPC elicited by atypical conclusions was larger than that from typical conclusions at parietal sites (*p* = 0.03). The three way interaction among frontality, laterality, and conclusion was significant. Further analysis indicated that the difference between general and atypical conclusions was found at F4 and FC4 (*ps* < 0.05), whereas the difference between typical and atypical conclusions was only found at P4 (*ps* < 0.01). No other difference was found.

We also analysed the link between behaviour and the evoked components of interest across subjects. The results found that there was no significant correlation between RTs and the mean amplitudes of N1, P2, N2, P3, and LPC for T-T, A-A, and G = G conditions. For the G-T, G-A, T-G, A-G, and A-T conditions, there were significant negative correlations between RTs and the LPC amplitudes at part of the frontal and frontal-central sites. The significant negative correlation between RTs and P2 amplitudes was only found for T-A condition at frontal sites. No other significant correlation was found.

## Discussion

The main goal of this study was to examine how types of premises modulate the typicality effect in category-based induction by focusing on the electrophysiological data elicited by the conclusions. The behaviour data showed that the processing of arguments with typical conclusions was faster than that of arguments with atypical ones in both general and typical premise conditions, but not in atypical premise conditions. Furthermore, the “strong” rate for arguments with typical conclusions was higher than that for atypical ones under all premise conditions. According to recent accounts^48^,^54^, the typicality was determined by the intercorrelation of semantic features. That is, the features that highly intercorrelated with other typical items were posed by typical members of the category relative to the atypical items. For example, sparrow and crow are typical birds because of their typical intercorrelated semantic features (*e.g*., wings, can fly), while ostrich is an atypical item because of its less intercorrelated features (*e.g*., cannot fly). In the present study, there are more common attributes between the premises and typical conclusions relative to atypical conclusions, resulting in higher inductive strength for typical items. These results were consistent with previous studies^1^,^4^, which suggested that arguments consisting of typical members can improve the acceptability of induction relative to arguments consisting of atypical members. One might suggest perhaps that typical items are more rapidly/easily processed than atypical items, leading to a greater ease (and more confidence) when people must make inferences about these items belonging to categories. However, such a view could not explain why no significant difference in RTs was found between typical and atypical conclusions in the atypical premise condition.

Furthermore, the “strong” rate for arguments with general conclusions was higher than atypical conclusions. A smaller P2 and N2, as well as a larger P3 and LPC at 500–600 ms, were elicited by general conclusions relative to atypical conclusions. These results are consistent with the research about the inclusion fallacy, in which generalisation for general conclusions is considered stronger than that for atypical conclusions in the specific premise conditions^1^,^55^. For example, Liang *et al*.^55^ found that the left fronto- temporal and superior medial frontal systems were specifically activated in response to fallacious responses (*e.g*., the argument such as “robins secrete uric acid crystals, therefore, birds secrete uric acid crystals” was considered more convincing than the argument that “robins secrete uric acid crystals, therefore, ostriches secrete uric acid crystals”).

The typicality effect was also present in the ERP data. As shown in Figs 2 and 3, when performing a category-based induction in general premise condition, a larger P2, as well as smaller P3 and LPC effects, were elicited by atypical conclusions relative to typical ones. The results in specific premise condition had different response patterns: larger P2 and LPC effects were found for atypical conclusions relative to typical conclusions in the typical premise condition, whereas only a larger P2 was found for atypical conclusions in the atypical premise condition. The divergence of P2, P3, and LPC effects for the typicality effect in general, and specific, premise conditions yielded a new insight into the neural underpinnings of the typicality effect in category-based induction task.

### N1 and P2 predicts perceptual processing and feature detection

Previous studies found a larger N1 effect for atypical stimuli relative to typical stimuli^6^,^12^,^13^. In fact, the N1 effect on the typicality effect was mainly found in studies using signal words or pictures as stimuli^12^,^13^. Although Lei *et al*.^6^ used a category-based deduction task, the conclusions consisted of only one word and a larger N1 was elicited by atypical ones. In the present study, a word was linked with a capital letter linked by a blank space to represent the argument, and this manipulation might reduce the difference in N1 effect. This might be the reason why, although the absolute N1 amplitude elicited by atypical conclusions (−1.85 μV) was larger than that for typical conclusions (−1.35 μV) in general premise conditions, the difference was not significant (*p*s > 0.23).

Similar to the findings of a previous study^6^, we also found a larger P2 effect for atypical conclusions relative to typical ones. As Lei *et al*.^6^ said, these results indicated that the typicality effect had a significant effect on the verification of an item’s category membership. In fact, research has found that the verification of typical members was faster than that of atypical ones^4^. As mentioned earlier, the P2 effect was modulated by higher-order perceptual processing^23^,^24^,^25^, as well as semantic processing and language^26^,^27^. In the present study, the detection of atypical items might involve more cognitive resources (*e.g*., attention processes increased with the organisational semantic demand), and elicited a larger P2 amplitude, just like the larger P2 elicited by Chinese characters with low combinability and consistency in orthographic and phonological processing^26^,^27^. This view can also be used to interpret the larger P2 effect for atypical conclusions relative to general conclusions, because the processing of the general category data at a basic level was faster than that for atypical members at subordinate level^56^.

### The P3 wave is specifically tuned to categorisation

Of greater interest was the observation of smaller P3 effect for atypical conclusion relative to typical conclusion in general premise conditions. As mention earlier, the P3 wave reflected the information-processing cascade related to attentional and memory mechanisms^23^,^31^,^32^. In the present study, the observed differences in P3 effect might reflect the attentional resource allocation needed for reasoning from general category to its typical or atypical members. That is, the smaller P3 effect indicated that the processing of atypical members involved more attentional resources during category-based induction. This view was further supported by the result that a larger P3 was elicited by general conclusion relative to typical and atypical conclusions, because the reasoning about general conclusions (especially in the G = G condition) was the simplest argument, which mobilised minimal cognitive resource and elicited the largest P3.

A related explanation was that the difference in P3 amplitude just reflected the satisfaction of expectations^19^,^34^,^35^,^36^,^37^, which was larger for matched conclusions than mismatched ones. In the general premise condition, a larger P3 amplitude was elicited by typical conclusions relative to atypical ones, because typical members have more features in common with the prototype induced by the category^57^,^58^. In fact, these results were consistent with data from studies during category-based verification, which found larger P3 peak amplitudes arising from typical items relative to atypical ones^11^. However, such differences in P3 amplitude between typical and atypical conclusions were not found in typical and atypical premise conditions. This may be because there was no significant difference in the expectations about the conclusions when reasoning under specific premise conditions. That is, different cognitive processes might be involved in reasoning with general and specific premises^1^,^59^. According to the similarity-based induction model, for example, the strength of the arguments was determined by the similarity and coverage processes^1^. Specifically, the similarity process refers to a calculation of the extent to which the premise categories are similar to the conclusion category, whereas the coverage process needs to calculate the degree of similarity between the premise categories and members of the lowest level category that includes both the premise and the conclusion categories. The induction using specific premises included both similarity and coverage processes, while only coverage process was involved in the general premise condition^1^. In the present study, additional processes (*e.g*., similarity) were involved in specific premise conditions, which reduced the effect of expectation or increased task difficulty, reducing the sensitivity of P3 thereto.

### LPC semantic content effects in the inference generation phase

More importantly, the LPC at 500–600 ms elicited by atypical conclusion was smaller than that arising from general and typical conclusions in general premise conditions, whereas the LPC at 600–700 ms elicited by atypical conclusion was larger than that for typical conclusions in typical premise conditions. Furthermore, no significant difference in LPC effect was found in atypical premise conditions. As mentioned earlier, LPC in a reasoning study reflected mismatched conclusions^19^,^35^,^37^, as well as the decision accuracy and confidence^46^.

In the general premise conditions, one plausible view was that the LPC effect among different conclusions reflected the old/new effect, as well as the contextual effect^42^,^43^. That is, for the G = G condition, same general category was presented in the premise and conclusion, and characterised by increases in LPC amplitude. Although the processing of typical members in the G-T condition was not repeated, participants could encode more information about the typical conclusion from the context at general category level, and elicited a larger LPC relative to that arising under atypical conclusions. Another plausible view was that the difference in LPC at 500–600 ms between typical and atypical conclusions was just a continuous P3 effect as mentioned above, which reflected attentional and memory allocation. In fact, the LPC at 500–600 ms elicited by atypical conclusions was also smaller than that arising from general conclusions in general premise conditions, which further supported this view.

In the typical premise condition, however, no item was repeated and the context of typical members could not lead to this difference. Similar to the findings of previous studies^35^,^37^, one plausible interpretation was that the LPC was more likely to reflect a mismatched conclusion. For example, research using deductive reasoning tasks has found that a larger LPC effect at 330–630 ms was elicited by a mismatched conclusion relative to that from a matched conclusion, which reflected the inference violation. In the present study, the mismatch in reasoning from typical premise to atypical conclusion was higher than reasoning from typical premise to typical conclusion, and thus elicited a larger LPC at 600–700 ms. Another plausible view was that the LPC at 600–700 ms reflected the processing manipulation, or decision accuracy and confidence^22^,^46^. That is, when participants were required to make inferences about different types of conclusion in the typical premise condition, the inductive strength of, or confidence in, atypical conclusions was smaller than that for typical conclusions, as they were not necessarily true and had different representatives. Or, to make a similar inductive inference, participants had to encode the atypical conclusion more deeply, which thus elicited a larger LPC.

The divergence of the LPC effect between typical and atypical conclusion in general and typical premise conditions might be caused by reasoning processing and the validity thereof. As mentioned earlier, the information about the conclusions in the general premise condition must be true, whereas in the typical premise condition they were not necessarily true. Furthermore, different processes (*e.g*., similarity and coverage) might be involved in the specific and general premise conditions, resulting in different LPC effects^1^. However, we did not dissociate the general premise from the specific premise by comparing ERP data directly. One reason was that previous studies have found significant differences in ERP waves elicited by categories with different hierarchical levels, such as a basic level, superordinate, and subordinate categories^60^,^61^. For example, ERP amplitudes between 320 and 420 ms reflected the differentiation between basic level and superordinate categorisations, whereas the dissociation of subordinate and basic level categorisations was mainly found at 450–550 ms^61^. As a result, it was difficult to determine whether, or not, the results were induced by conceptual processing, or reflected inference processing, even though we found significant differences between general and specific premises (*e.g*., LPC). Future research into this issue was deemed necessary.

More generally, diverging evidence was found for the typicality effect in category-based induction under different premise conditions. That is, although P2 has a stable reaction to the typicality effect under all premise conditions, the P3 and LPC at 500–600 ms were only sensitive to the typicality effect under general premise conditions, and the LPC at 600–700 ms was only found for the typicality effect under typical premise conditions. These results are consistent with the dual-process accounts of reasoning, which posited that there are two distinct cognitive systems underlying reasoning^62^,^63^. Specifically, system 1 was largely fast, automatic, unconscious, and competed with an analytic system, whereas system 2 was slower, deliberative, conscious, and based on rules. In the present study, the stable differences in P2 effect might be related to system 1, whereas the diverging evidence pertaining to P3 and LPC were related to system 2. By manipulating the premise-conclusion similarity (or argument length) and logical validity of arguments, recent studies indicated that a two-process account of reasoning was more suited to interpretation of the relationships between inductive reasoning and deductive reasoning^17^,^18^. For example, for a common set of arguments, induction judgments (“strong” or “not strong”) were more affected by the premise-conclusion similarity, whereas deduction judgments (“valid” or “not valid”) were more affected by validity^1^,^17^. In the present study, the typicality might be another qualitative phenomenon that could be used to support the two-process account of reasoning, just like the premise-conclusion similarity^17^. However, only induction judgments were included in the present study, and further study including deduction judgments might help to shed light on this issue. As the spatial resolution of the ERP is rather low, any future study should combine the fMRI recordings with ERP recordings to explore this issue further.

## Conclusion

The present findings have yielded new insights into the processing of typicality effect in a category-based induction by manipulating the types of premise and conclusion. The larger P2 effect for atypical conclusions relative to typical conclusions, which was found in all three premise conditions, served as markers of typicality processing and features detection. The larger P3 effect for typical conclusions relative to atypical conclusions, which was found only in general premise condition, indicated that the P300 amplitude could reflect resource allocation. Furthermore, a larger LPC effect at 500–600 ms was elicited by typical conclusions relative to atypical conclusions in general premise conditions, which reflected the contextual effect between a category and its members, or just a continuous P3. However, the LPC at 600–700 ms elicited by typical conclusions was smaller than that arising from atypical conclusions, which is more likely to reflect inference violation or processing manipulation. Overall, these results suggested that the types of premises had critical regulatory roles in the typicality effect in category-based induction, which reflected on the P2, P3, and LPC effects.

## Additional Information

**How to cite this article**: Liang, X. *et al*. How types of premises modulate the typicality effect in category-based induction: diverging evidence from the P2, P3, and LPC effects. *Sci. Rep.* **6**, 37890; doi: 10.1038/srep37890 (2016).

**Publisher’s note:** Springer Nature remains neutral with regard to jurisdictional claims in published maps and institutional affiliations.

## Supplementary Material

Supplementary Information

## Figures and Tables

**Table 1:** The main conditions and examples used in the experiment.

Conditions	Arguments	Premise	Conclusion	Properties	Examples	
					Premise	Conclusion
General premise condition	G = G	General	General	Same	Fruits C	Fruits C?
	G-T	General	Typical	Same	Fruits A	Apple A?
	G-A	General	Atypical	Same	Fruits A	Raspberry A?
Typical premise condition	T-G	Typical	General	Same	Apple A	Fruits A?
	T-T	Typical	Typical	Same	Peach B	Orange B?
	T-A	Typical	Atypical	Same	Litchi B	Raspberry B?
Atypical premise condition	A-G	Atypical	General	Same	Raspberry A	Fruits A?
	A-T	Atypical	Typical	Same	Raspberry B	Litchi B?
	A-A	Atypical	Atypical	Same	Raspberry B	Guava B?
Baseline conditions	T = T	Typical	Typical	Same	Apple A	Apple A?
	A = A	Atypical	Atypical	Same	Guava B	Guava B?
	Control	Natural	Artificial	Different	Apple C	Piano B?

**Table 2:** Four-way repeated-measures ANOVA of mean amplitudes to assess the effects of typicality on category-based induction.

Four-way ANOVA	N1			P2			N2			P3			LPC								
	(50–150 ms)			(150–250 ms)			(250–350 ms)			(350–450 ms)			(500–600 ms)			(600–700 ms)			(700–800 ms)		
	*F*	*p*	*η*^2^	*F*	*p*	*η*^2^	*F*	*p*	*η*^2^	*F*	*p*	*η*^2^	*F*	*p*	*η*^2^	*F*	*p*	*η*^2^	*F*	*p*	*η*^2^
Frontality	11.95	***0.002***	0.46	0.25	0.662	0.02	13.02	***0.001***	0.48	23.07	***0.000***	0.62	27.28	***0.000***	0.66	14.27	***0.000***	0.51	7.19	***0.005***	0.34
Laterality	12.18	***0.000***	0.47	4.28	***0.030***	0.23	19.52	***0.000***	0.58	10.93	***0.001***	0.44	9.40	***0.002***	0.40	8.64	***0.004***	0.38	10.57	***0.002***	0.43
Premise	1.36	0.274	0.09	0.43	0.637	0.03	5.10	***0.027***	0.27	6.96	***0.007***	0.33	8.10	***0.003***	0.37	6.68	***0.004***	0.32	3.66	***0.047***	0.21
Conclusion	0.33	0.692	0.02	11.71	***0.000***	0.46	9.36	***0.002***	0.40	10.87	***0.001***	0.44	0.85	0.435	0.06	0.44	0.587	0.03	0.56	0.529	0.04
Frontality* Laterality	7.99	***0.000***	0.36	6.74	***0.001***	0.33	2.13	0.100	0.13	4.52	***0.007***	0.24	8.19	***0.000***	0.37	8.55	***0.000***	0.38	8.60	***0.000***	0.38
Frontality*|premise	0.66	0.563	0.05	0.19	0.893	0.01	2.24	.102	0.14	0.81	0.472	0.06	1.41	0.254	0.09	1.87	0.143	0.12	2.07	0.106	0.13
laterality*premise	3.74	***0.016***	0.21	0.88	0.463	0.06	3.42	***0.021***	0.20	3.66	***0.018***	0.21	3.80	***0.014***	0.21	3.20	***0.025***	0.19	1.34	0.273	0.09
Frontality* Laterality*premise	1.15	0.342	0.08	1.40	0.226	0.09	0.94	0.467	0.06	0.73	0.621	0.05	0.91	0.495	0.06	1.12	0.359	0.07	0.96	0.461	0.06
Frontality* Conclusion	0.63	0.584	0.04	4.20	***0.017***	0.23	0.68	0.537	0.05	0.34	0.777	0.02	1.71	0.182	0.11	3.99	***0.019***	0.22	3.57	***0.028***	0.20
laterality* Conclusion	1.71	0.180	0.11	1.73	0.191	0.11	14.68	***0.000***	0.51	6.06	***0.001***	0.30	4.55	0.008	0.25	3.69	***0.021***	0.21	2.13	0.122	0.13
Frontality* Laterality*Conclusion	2.06	0.069	0.13	4.87	***0.001***	0.26	3.41	***0.008***	0.20	2.89	***0.013***	0.17	3.09	0.006	0.18	3.65	***0.003***	0.21	2.39	***0.035***	0.15
Premise*Conclusion	1.29	0.292	0.08	1.45	0.240	0.09	3.12	***0.038***	0.18	5.97	***0.002***	0.30	3.39	0.023	0.20	2.83	***0.047***	0.17	0.71	0.559	0.05
Frontality* Premise*Conclusion	2.72	0.044	0.16	0.97	0.434	0.07	4.86	***0.004***	0.26	4.68	***0.002***	0.25	2.08	0.089	0.13	0.41	0.805	0.03	0.84	0.511	0.06
Laterality* Premise*Conclusion	1.82	0.121	0.12	1.75	0.143	0.11	5.05	***0.001***	0.27	3.02	***0.024***	0.18	2.94	0.027	0.17	1.75	0.147	0.11	0.43	0.802	0.03
Frontality* Laterality* Premise*Conclusion	1.83	0.079	0.12	2.58	***0.009***	0.16	0.88	0.543	0.06	1.32	0.240	0.09	1.06	0.397	0.07	1.05	0.405	0.07	0.94	0.483	0.06

**Table 3:** Four-way repeated-measures ANOVA of mean peak latency and amplitude to assess the effects of typicality on category-based induction.

Four-way ANOVA	Peak latency												Peak amplitude											
	N1			P2			N2			P3			N1			P2			N2			P3		
	(50–150 ms)			(150–250 ms)			(250–350 ms)			(350–450 ms)			(50–150 ms)			(150–250 ms)			(250–350 ms)			(350–450 ms)		
	*F*	*p*	*η*^*2*^	*F*	*p*	*η*^*2*^	*F*	*p*	*η*^*2*^	*F*	*p*	*η*^*2*^	*F*	*p*	*η*^*2*^	*F*	*p*	*η*^*2*^	*F*	*p*	*η*^*2*^	*F*	*p*	*η*^*2*^
Frontality	1.11	0.323	0.07	30.50	***0.000***	0.69	5.29	*0.020*	0.27	1.01	0.384	0.07	7.73	***0.007***	0.36	0.57	0.499	0.04	12.81	***0.001***	0.48	16.33	***0.000***	0.54
Laterality	0.45	0.629	0.03	6.73	***0.006***	0.33	0.40	0.582	0.03	0.41	0.614	0.03	6.15	***0.011***	0.31	4.13	***0.031***	0.23	18.38	***0.000***	0.57	9.82	***0.001***	0.41
Premise	1.29	0.292	0.08	0.90	0.404	0.06	0.73	0.468	0.05	1.14	0.334	0.08	4.05	***0.039***	0.22	0.40	0.632	0.03	5.09	***0.022***	0.27	7.29	***0.007***	0.34
Conclusion	1.26	0.298	0.08	0.26	0.742	0.02	13.60	*0.000*	0.49	3.22	0.057	0.19	0.54	0.554	0.04	17.61	***0.000***	0.56	5.50	***0.017***	0.28	13.94	***0.000***	0.50
Frontality* Laterality	0.79	0.474	0.05	1.80	.153	0.11	1.03	0.406	0.07	0.96	0.437	0.06	1.66	0.194	0.11	12.59	***0.000***	0.47	1.33	0.277	0.09	2.18	0.105	0.14
Frontality*premise	0.86	0.500	0.06	0.78	0.538	0.05	0.48	0.733	0.03	0.66	.586	0.05	0.12	0.939	0.01	0.62	0.654	0.04	1.44	0.250	0.09	1.70	0.185	0.11
laterality*premise	3.84	***0.024***	0.22	1.74	.178	0.11	1.53	0.222	0.10	3.74	***0.015***	0.21	2.75	0.065	0.16	0.84	.471	0.06	1.56	0.212	0.10	2.52	0.063	0.15
Frontality* Laterality*premise	2.20	0.053	0.14	0.63	0.686	0.04	0.91	0.488	0.06	0.92	0.498	0.06	1.90	0.089	0.12	0.85	0.505	0.06	1.02	0.414	0.07	0.49	0.818	0.03
Frontality* Conclusion	1.74	0.177	0.11	1.57	0.199	0.10	1.38	0.257	0.09	0.63	0.614	0.04	0.45	0.702	0.03	2.11	0.115	0.13	0.47	0.643	0.03	0.73	0.525	0.05
laterality* Conclusion	0.72	0.542	0.05	0.61	0.598	0.04	3.96	*0.018*	0.22	0.84	0.474	0.06	1.18	0.326	0.08	2.25	0.121	0.14	5.26	***0.004***	0.27	4.79	***0.006***	0.26
Frontality* Laterality*Conclusion	0.69	0.643	0.05	1.62	0.150	0.10	1.66	0.161	0.11	1.39	0.224	0.09	5.17	***0.000***	0.27	2.74	***0.020***	0.16	3.07	***0.012***	0.18	1.47	0.185	0.10
Premise*Conclusion	1.24	0.309	0.08	3.98	***0.012***	0.22	0.81	0.494	0.06	0.29	0.835	0.02	1.10	0.357	0.07	0.69	0.573	0.05	2.03	0.132	0.13	6.11	***0.004***	0.30
Frontality* Premise*Conclusion	0.70	0.584	0.05	1.14	0.348	0.08	2.01	0.073	0.13	1.23	0.299	0.08	2.25	0.079	0.14	0.72	0.619	0.05	2.39	0.074	0.15	4.74	***0.001***	0.25
Laterality* Premise*Conclusion	0.74	0.564	0.05	0.95	0.440	0.06	1.33	0.271	0.09	0.62	0.663	0.04	1.08	0.380	0.07	1.26	0.300	0.08	4.48	***0.004***	0.24	1.94	0.109	0.12
Frontality* Laterality* Premise*Conclusion	1.14	0.347	0.08	1.13	0.354	0.07	0.65	0.733	0.04	1.42	0.195	0.09	1.45	0.192	0.09	1.49	0.181	0.10	0.71	0.659	0.05	1.58	0.137	0.10
