# Supplementary material for: How types of premises modulate the typicality effect in category-based induction: diverging evidence from the P2, P3, and LPC effects
Source: Sci Rep. 2016 Dec 16;6:37890. doi: 10.1038/srep37890 (PMC5159785; doi:10.1038/srep37890)
Supplement: Supplementary Information [file srep37890-s1.doc]

**How types of premises modulate the typicality effect in category-based induction: diverging evidence from the P2, P3, and LPC effects**

Xiuling Liang^1^, Qingfei Chen^12^*, Yi Lei^1^, Hong Li^1^*

*(*^1^ *Research Centre for Brain Function and Psychological Science, Shenzhen University, Shenzhen 518060, China)*

*(*^2^ *China center for special economic zone research, Shenzhen University, Shenzhen 518060, China)*

Address Correspondence to: Qingfei Chen, Hong Li.

Research Centre for Brain Function and Psychological Science, Shenzhen University, Shenzhen 518060

E-mail address: myth3233@126.com (Q. Chen); lihongwrm@vip.sina.com (H. Li).

Tel: +86 755 26534423.

**Highlights**

- We investigate the modulation of premises on typicality effect in category-based induction
- Only P2 effect was sensitive to typicality effect in atypical premise condition
- Both P2 and LPC at 600-700ms were sensitive to typicality effect in typical premise condition
- Typicality effect reflects on P2, P3 and LPC at 500-600 ms in the general premise condition
- The typicality effect in different premises involves different cognitive processes

**S1 Table. Normed Natural Categories (Animal) Used in the Experiments.**

| Categories | English translations | Typicality scores | Categories | English translations | Typicality scores | Categories | English translations | Typicality scores |
| --- | --- | --- | --- | --- | --- | --- | --- | --- |
| 鱼 | fish |  | 鸟 | bird |  | 昆虫 | insect |  |
| 鲫鱼 | carp | 6.9 | 麻雀 | sparrow | 6.8 | 蜜蜂 | bee | 6.7 |
| 草鱼 | grass carp | 6.9 | 乌鸦 | crow | 6.7 | 蜘蛛 | spider | 6.6 |
| 青鱼 | herring | 6.8 | 鹦鹉 | parrot | 6.7 | 蜻蜓 | dragonfly | 6.5 |
| 黄花鱼 | croaker | 6.8 | 燕子 | swallow | 6.7 | 蝴蝶 | butterfly | 6.5 |
| 鲶鱼 | catfish | 6.8 | 喜鹊 | magpie | 6.7 | 蝗虫 | locust | 6.5 |
| 黄鳝 | Eel | 4.7 | 鸸鹋 | emu | 4.8 | 跳蚤 | flea | 5 |
| 泥鳅 | loach | 4.5 | 鸭子 | duck | 4.7 | 蜉蝣 | ephemera | 4.8 |
| 蝠鲼 | manta | 4.3 | 鸵鸟 | ostrich | 4.4 | 豆娘 | damsel-fly | 4.8 |
| 海龙 | sea dragon | 3.7 | 企鹅 | penguin | 4.2 | 纺织娘 | katydid | 4.7 |
| 海马 | seahorse | 3.6 | 鸡 | chicken | 4.1 | 虱子 | louse | 4.5 |

**S2 Table. Normed Natural Categories (Plant) Used in the Experiments.**

| Categories | English translations | Typicality scores | Categories | English translations | Typicality scores | Categories | English translations | Typicality scores |
| --- | --- | --- | --- | --- | --- | --- | --- | --- |
| 蔬菜 | vegetables |  | 花 | flower |  | 水果 | fruit |  |
| 白菜 | cabbage | 6.8 | 康乃馨 | carnation | 7 | 苹果 | apple | 7 |
| 茄子 | eggplant | 6.8 | 牡丹 | peony | 7 | 桃子 | peach | 7 |
| 苦瓜 | balsam pear | 6.7 | 牡丹 | peony | 6.9 | 西瓜 | watermelon | 7 |
| 菠菜 | spinach | 6.7 | 梅花 | plum flower | 6.9 | 橘子 | orange | 6.9 |
| 芹菜 | celery | 6.7 | 荷花 | lotus | 6.9 | 荔枝 | litchi | 6.8 |
| 木耳 | fungus | 6 | 夕颜 | moonflower | 5 | 山竹 | mangosteen | 5.4 |
| 葫芦 | gourd | 5.7 | 紫云英 | milk vetch | 4.8 | 红毛丹 | rambutan | 5.2 |
| 魔芋 | konjac | 4.9 | 扶桑 | hibiscus rosa-sinensis | 4.7 | 番石榴 | guava | 5 |
| 海带 | kelp | 4.2 | 辛夷 | magnolia | 4.6 | 雾莲 | fog lin | 4.3 |
| 秋葵 | okra | 4.1 | 玉簪 | Jade hairpin | 4.4 | 覆盆子 | raspberry | 4.3 |

**S3 Table. Normed Artificial Categories Used in the Experiments.**

| Categories | English translations | Typicality | Categories | English translations | Typicality | Categories | English translations | Typicality | Categories | English translations | Typicality |
| --- | --- | --- | --- | --- | --- | --- | --- | --- | --- | --- | --- |
| 衣物 | clothing |  | 乐器 | musical instruments |  | 家具 | furniture |  | 工具 | tool |  |
| 衬衣 | shirt | 6.8 | 古筝 | the zither | 6.8 | 沙发 | sofa | 7 | 锤子 | hammer | 6.8 |
| 运动衫 | Sweatshirt | 6.8 | 钢琴 | piano | 6.8 | 床 | bed | 7 | 扳手 | wrench | 6.7 |
| 毛衣 | Sweater | 6.7 | 吉他 | guitar | 6.8 | 茶几 | coffee table | 6.9 | 起子 | screwdriver | 6.7 |
| 牛仔裤 | jeans | 6.7 | 二胡 | erhu | 6.8 | 餐桌 | dining table | 6.9 | 剪刀 | scissors | 6.7 |
| 西服 | Suit | 6.6 | 小提琴 | violin | 6.7 | 椅子 | chair | 6.8 | 钳子 | pliers | 6.6 |
| 汉服 | Chinese clothing | 5.2 | 磬 | chime stone | 5.1 | 婴儿床 | infanette | 5.6 | 铣刀 | cutter | 5.4 |
| 袍子 | robe | 5.1 | 阮 | nguyen | 5.1 | 五斗橱 | chest of drawers | 5.6 | 卡尺 | caliper | 5.4 |
| 斗篷 | cloak | 4.8 | 埙 | Xun | 5 | 屏风 | screen | 5.1 | 砂轮 | grinding wheel | 5.2 |
| 纱丽 | Sari | 4.6 | 箜篌 | konghou | 4.8 | 条案 | reads | 4.8 | 车床 | lathe | 5 |
| 盔甲 | armor | 3.8 | 筑 | zhu | 4.4 | 榻榻米 | tatami | 4.7 | 轴承 | bearing | 4.6 |
